# Criterion Validity of the Implicit Positive and Negative Affect Test: Prediction of Facial Affect Perception

**DOI:** 10.3389/fpsyg.2021.635368

**Published:** 2021-10-01

**Authors:** Anna-Sophie Weil, Vivien Günther, Frank Martin Schmidt, Anette Kersting, Markus Quirin, Thomas Suslow

**Affiliations:** ^1^Department of Psychosomatic Medicine and Psychotherapy, University of Leipzig Medical Centre, Leipzig, Germany; ^2^Department of Child and Adolescent Psychiatry, Psychotherapy and Psychosomatics, University of Leipzig Medical Centre, Leipzig, Germany; ^3^Department of Psychiatry and Psychotherapy, University of Leipzig Medical Centre, Leipzig, Germany; ^4^School of Management, TU München, Munich, Germany; ^5^Department of Psychology, PFH Göttingen, Göttingen, Germany

**Keywords:** implicit positive and negative affect test (IPANAT), implicit affect, explicit affect, perception of facial emotion, criterion validity

## Abstract

This study focused on the criterion-related validity of the Implicit Positive and Negative Affect Test (IPANAT). The IPANAT is thought to be a measure of automatic activation of cognitive representations of affects. In this study, it was investigated whether implicit affect scores differentially predict ratings of facial emotions over and above explicit affectivity. Ninety-six young female participants completed the IPANAT, the Positive and Negative Affect Schedule (PANAS) as an explicit measure of state and trait affectivity, and a task for the perception of facial emotions. Implicit negative affect predicted the perception of negative but not positive facial emotions, whereas implicit positive affect predicted the perception of positive but not negative facial emotions. The observed double-dissociation in the correlational pattern strongly supports the validity of the IPANAT as a measure of implicit affectivity and is indicative of the orthogonality and thus functional distinctness of the two affect dimensions of the IPANAT. Moreover, such affect-congruent correlations were absent for explicit affect scales, which additionally supports the incremental validity of the IPANAT.

## Introduction

For a long time, psychological research has been struggling with measuring affects without tapping into problems associated with explicit assessment. Explicit or self-report measures, which assess affectivity directly, are biased by factors, such as social desirability, impression management strategies, and reduced introspective ability (Mitchell and Tetlock, 2015). To avoid such difficulties and to make other facets of the construct accessible, Quirin et al. (2009a) developed the Implicit Positive and Negative Affect Test (IPANAT). Explicit affect is formed by conscious reflections and refers to conscious affective experiences (Quirin and Bode, 2014).

The IPANAT was designed to measure implicit affect, defined as the automatic activation of cognitive representations of affective experiences. In this test, participants are instructed to provide ratings concerning the degree to which artificial words from a putative artificial language express a number of affective states or traits (Quirin et al., 2018). The IPANAT makes use of the principle of affect infusion according to which affect exerts an impact on evaluative processes influencing affective perceptions of unrelated objects (Forgas, 2001). In-line with two-dimensional models of affect (Watson et al., 1988), positive affect and negative affect are treated as two different dimensions in the IPANAT. The IPANAT scales have shown relationships of small-to-moderate strength with explicit measures of the same affect type (Quirin et al., 2009a). In study 2 of Quirin et al. (2009a), the IPANAT scales correlated with tests and tasks assessing aspects of implicit affectivity, the Neutral Objects Satisfaction Questionnaire that requires ratings on satisfaction with everyday objects (Judge and Bretz, 1993), and a memory retrieval task of affective contents based on completion of word fragments (Graf et al., 1982). These findings provide important indications of the validity of the IPANAT as a measure of implicit affect.

In this study, aspects of criterion-related validity were investigated by correlating the IPANAT scales with judgments of facial emotions perceived. Criterion-related validity refers to the degree to which a measurement can predict an outcome for another measure (Cohen and Swerdlik, 2005). A test has criterion validity if it is useful for accurately predicting performance or behavior in another (past, present, or future) situation. We used schematic faces with different expressions to assess the perception of emotions in faces (Bouhuys et al., 1997). The German version of the Positive and Negative Affect Schedule (PANAS; Krohne et al., 1996), which is a self-report questionnaire, was administered to assess explicit positive and negative state and trait affects (i.e., actual, persistent, and habitual affects). Based on previous findings concerning the implicit processing of lexical material (see Quirin et al., 2009a, study 2), it was hypothesized that implicit but not explicit affect scores (i.e., positive, and negative state and trait affect as assessed by the PANAS) predict judgments of facial emotions. Specifically, we expected that implicit positive affect is associated with enhanced attribution of positive affect, and implicit negative affect is associated with heightened attribution of negative affect, regardless of the type of affective expression. We also examined the relationships of IPANAT scales with explicit state and trait affect as assessed by the PANAS.

## Materials and Methods

### Participants

Participants were recruited using social media and announcements in buildings of the university. Most of the participants were university students, and all of them were women. Exclusion criteria were a lifetime history of psychiatric or neurological diseases, psychotropic medication use, and no native German speaking. The present sample consisted of 96 women with a mean age of 23.9 years (SD = 3; range: 19–30). School education of the participants had a mean duration of 12.3 years (SD = 0.6), and the mean verbal IQ as assessed by the Mehrfachwahl-Wortschatz-Intelligenz test (MWT-B) was 112.1 (SD = 10.7). Our study was carried out in accordance with the code of ethics (Declaration of Helsinki) of the World Medical Association (2013). Our study was approved by the ethics committee at the University of Leipzig, Medical Faculty (reference number 138-14-14042014). All participants gave written informed consent after reading a description of the study and received financial compensation.

### Questionnaires and Tests

Implicit positive and negative affects were measured by the IPANAT (Quirin et al., 2009a). The IPANAT assesses affect in an indirect way by asking participants to rate the extent to which artificial words (e.g., VIKES) from a putative artificial language express a certain mood on a four-point scale. Each of the artificial words is presented along with three positive and three negative affective words. For assessing explicit positive and negative state and trait affects, the German version of the PANAS (Krohne et al., 1996) was used. The PANAS consists of 20 adjectives, 10 describing positive and the other 10 negative affective states. On a five-point scale, the participants rated how much they feel like this at the moment (state version) or in general (trait version). Verbal intelligence was assessed by the MWT-B (Lehrl, 2005).

### Perception of Facial Emotions

**Figure 1:**
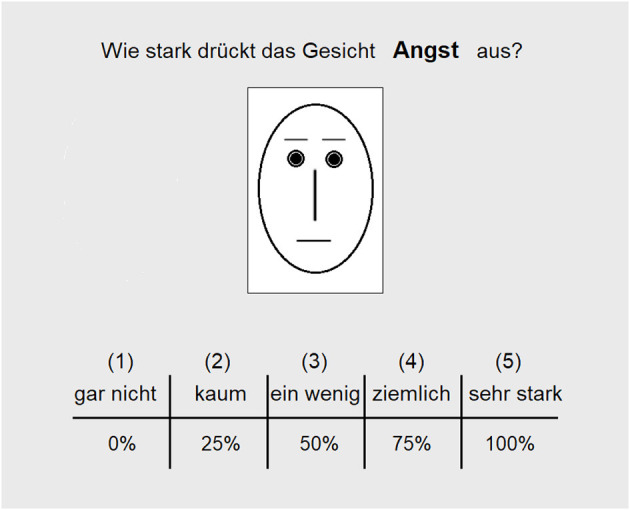
In the perception of facial expressions (PFE) task, schematic faces were displayed. In the present example, a face with a neutral expression is shown. Images of faces remained on the screen until response. Participants had to evaluate the face with respect to the degree to which it expresses a certain affect (in the example the question is “How strongly does the face express fear?”). At the bottom of the screen, the response format was shown. Participants evaluated the expression of seven affects (positive: happiness, attraction; negative: fear, anger, sadness, disgust, and rejection). Answers were given on a keyboard by pressing the “1”, “2”, “3”, “4”, or “5” key.

The perception of facial expressions (PFE) task consisted of 12 schematic oval faces as applied by Bouhuys et al. (1997). The line drawings were composed of one type of eyes and nose, four eyebrows, and three mouth types (see Bouhuys et al., 1997, for stimulus material). The schematic faces had negative, positive, neutral, and ambiguous expressions. Images of faces remained on the screen until a response was given. To control stimulus presentation and record performance, Presentation software (Version 16.3, Neurobehavioral Systems, Berkeley, CA, USA; www.neurobs.com) was used. Participants were instructed to evaluate schematic drawings of faces with respect to the degree to which they express certain affects. At the top of the screen, a question (e.g., “How strongly does the face express sadness?”) was presented. At the bottom of the screen, the response format was shown. Responses were given on a five-point scale ranging from 0% (*not at all, 1*), 25% (*very little, 2*), 50% (*little, 3*), and 75% (*quite strongly, 4*) to 100% (*very strongly, 5*), while the randomly selected facial stimuli appeared in the center of the screen. Following Bouhuys et al. (1997), subjects had to evaluate with respect to seven basic or relational affects [*positive:* happiness (Freude), attraction (Anziehung); *negative:* fear (Angst), anger (Ärger), sadness (Traurigkeit), disgust (Ekel), and rejection (Zurückweisung) (see also Hale et al., 1998)]. In total, participants made 84 judgments in the PFE task (12 faces × 7 affect ratings). Answers were given on a keyboard by pressing the “1”, “2”, “3”, “4”, or “5” key. In Figure 1, we show an example of a trial of the PFE task. Two cumulative scores were calculated from the evaluative PFE data: (1) Judgments of the 5 negative categories were averaged over the 12 facial stimuli, and (2) judgments of the 2 positive categories were averaged over the 12 facial stimuli.

### General Procedure

Testing sessions were conducted individually in a quiet room free from auditory and visual distractions. Tests were administered in a fixed order with participants starting to complete the IPANAT and the PANAS state scales and proceeding with the PFE task. A laptop (Dell Latitude E6510) was used for the presentation of the PFE task. Thereafter, participants filled out the PANAS trait scales and the MWT-B.

### Statistical Analysis

Product moment correlation analyses were performed to explore the relationships between measures of affectivity, intelligence, and PFE. In addition, hierarchical regression analyses were conducted for perceived positive affect and perceived negative affect. In the first step, explicit positive and explicit negative affects (state and trait) as assessed by the PANAS and intelligence (IQ scores) were entered as predictors. The second step included entering implicit positive affect and implicit negative affect as measured by the IPANAT. Results were considered significant at *p* < 0.05, two-tailed. All calculations were administered using SPSS 25.0 (IBM Corp., Armonk, NY, USA).

## Results

As expected, implicit positive affect (IPANAT-PA) showed a positive correlation with the PANAS-PA state, whereas implicit negative affect (IPANAT-NA) was not correlated with any of the PANAS scores (see Table 1). Moreover, the PANAS-PA state showed a correlation with the PANAS-PA trait. PANAS-NA state was correlated with the PANAS-NA trait. Negative correlations of PANAS-NA state were observed with PANAS-PA state and PANAS-PA trait. Intelligence was correlated only with the PANAS-PA trait. IPANAT-PA correlated positively with the perception of positive affect in facial expressions. In addition, a positive correlation between the PANAS-NA trait and the perception of positive affect was revealed. Finally, IPANAT-NA correlated positively with the perception of negative affect (see Table 1).

**Table 1:** Descriptive statistics and correlations between psychological measures.

	**1**	**2**	**3**	**4**	**5**	**6**	**7**	**8**	**9**
**1**. IPANAT-PA	–								
**2**. IPANAT-NA	−0.13	–							
**3**. PANAS-PA state	0.30^**^	0.00	–						
**4**. PANAS-PA trait	0.16	−0.05	0.59^***^	–					
**5**. PANAS-NA state	0.03	0.10	−0.23^*^	−0.25^*^	–				
**6**. PANAS-NA trait	0.13	0.10	−0.16	−0.11	0.39^***^	–			
**7**. MWT-B IQ	0.06	−0.02	0.17	0.29^**^	−0.12	−0.04	–		
**8**. PFE Positive affect	0.34^***^	0.12	0.13	0.18	0.11	0.26^**^	0.02	–	
**9**. PFE Negative affect	0.01	0.32^**^	−0.02	0.00	0.14	0.18	−0.18	0.02	–
Mean	2.39	1.84	33.32	35.29	13.03	15.06	112.1	2.05	2.44
SD	0.37	0.34	5.54	5.63	3.63	3.91	10.7	0.27	0.34

* *p < 0.05*;

** *p < 0.01*;

*** *p < 0.001 (two-tailed)*.

*IPANAT-PA, Implicit Positive and Negative Affect Test, implicit positive affect; IPANAT-NA, Implicit Positive and Negative Affect Test, implicit negative affect; PANAS-PA, Positive Affect Scale of the Positive and Negative Affect Schedule; PANAS-NA, Negative Affect Scale of the Positive and Negative Affect Schedule; MWT-B IQ, Multiple-choice vocabulary test version B intelligence quotient; PFE Positive affect, mean perceived positive affect in the Perception of facial expressions task; PFE Negative affect, mean perceived negative affect in the Perception of facial expressions task*.

Two hierarchical regression analyses were conducted (for perceived positive affect and perceived negative affect as dependent variables). The regression model concerning the perception of positive affect yielded in the first step (which included PANAS-PA state and trait, PANAS-NA state and trait, and verbal IQ as measured by the Mehrfachwahl-Wortschatz-Intelligenz test [MWT-B]) a significant result: Explicit negative trait affect was a significant predictor of positive affect perception (see Table 2). In the second step, after the inclusion of IPANAT-PA and -NA, implicit positive affect was found to significantly predict perceived positive affect in facial expressions (see Table 2). The first step of the regression analysis regarding the perception of negative affect did not show a significant result. In the second step, after the inclusion of IPANAT-PA and -NA, implicit negative affect predicted the perception of negative emotions in faces (see Table 2).

**Table 2:** Hierarchical multiple regression analyses with perceived affect in the PFE task as dependent variables and affect measures and MWT-B as predictors.

	**Perceived Positive Affect**			**Perceived Negative Affect**		
	**β**	**R** ^**2**^	**ΔR** ^**2**^	**β**	**R** ^**2**^	**ΔR** ^**2**^
**Step 1**		0.12	0.12^*^		0.07	0.07
PANAS-PA state	0.084			−0.003		
PANAS-PA trait	0.189			0.094		
PANAS-NA state	0.070			0.080		
PANAS-NA trait	0.271^*^			0.149		
MWT-B IQ	−0.032			−0.188		
**Step 2**		0.21	0.09^**^		0.16	0.09^*^
IPANAT-PA	0.306^**^			0.037		
IPANAT-NA	0.146			0.308^**^		

* *p < 0.05*;

** *p < 0.01*.

*PANAS-PA, Positive Affect Scale of the Positive and Negative Affect Schedule; PANAS-NA, Negative Affect Scale of the Positive and Negative Affect Schedule; MWT-B IQ, Multiple-choice vocabulary test version B intelligence quotient; IPANAT-PA, Implicit Positive and Negative Affect Test, implicit positive affect; IPANAT-NA, Implicit Positive and Negative Affect Test, implicit negative affect; Perceived positive affect, mean perceived positive affect in the Perception of facial expressions task; Perceived negative affect, mean perceived negative affect in the Perception of facial expressions task*.

## Discussion

For this study, we expected that implicit but not explicit affect predicts ratings of facial emotions. Supporting this hypothesis, we found that implicit negative affect was the only variable predicting the perception of negative affect in schematic faces. Moreover, IPANAT-PA predicted the attribution of positive affect to faces, independent of explicit affect. The observed correlations with face perceptions support the criterion-related validity of the IPANAT as a measure of implicit affectivity complementing and extending previous work based on the processing of lexical material (Quirin et al., 2009a, study 2). Importantly, in our investigation, participants were not asked to describe the way the facial expressions make them feel but they had to judge how strongly the faces express positive or negative emotions. In study 2 of Quirin et al. (2009a), it was shown that implicit affect as assessed by the IPANAT is related to the degree to which positive and negative contents or experiences are retrieved from memory. These findings are in-line with the conceptualization of implicit affect as automatic activation of affect representations, which influences memory retrieval and judgmental processes. The correlation results obtained reveal incremental predictive validity of the IPANAT suggesting that this measure can provide important information on how people assess and interpret social signals, above and beyond explicit affect measures. Finally, the present data show additional validity for the functional distinctness of the two affect dimensions of the IPANAT.

In our study, an unexpected correlation was found between explicit negative trait affect and perceived positive affect. This could mean that heightened self-reported habitual negative affect goes along with an increased perception of positive affect in facial expressions. This correlation might result from mood repair efforts in our healthy subjects. There is evidence from eye-tracking studies that healthy individuals increase their attention to positive images and happy faces to counteract and repair sad or negative moods (Sanchez et al., 2014; Newman and Sears, 2015). Possibly, a positive bias in the affective perception of the facial expressions of others may in the long run contribute to a reduction in negative affect experienced in everyday life.

Evidence for criterion and construct validity of the IPANAT has also been provided by recent neuropsychological and neurobiological studies. It was shown that implicit affect as assessed by the IPANAT predicts attention bias to negative faces when looking at crowds of faces (Bodenschatz et al., 2018), recognition of briefly presented angry body posture, and neural activation of subcortical structures in response to threatening body expressions (Suslow et al., 2015). Moreover, implicit affect as measured by the IPANAT has been found to be a predictor of autonomous nervous system reactions, such as stress-related cardiovascular activity (van der Ploeg et al., 2016) and cortisol secretion (Quirin et al., 2009b), beyond explicit methods of affect assessment (see Weil et al., 2019, for a review). Proof for cross-cultural validity of the IPANAT emerged recently (Sulejmanov and Spasovski, 2017; Quirin et al., 2018; Hernández et al., 2020b). It should be noted that a short form of the IPANAT consisting only of 18 items, the IPANAT-18, has been developed. The IPANAT-18 showed satisfactory psychometric properties and seemed to represent a promising tool for economically measuring implicit affectivity (Hernández et al., 2020a).

In our study, we also examined the correlations of the IPANAT scales with explicit state and trait affect measures. According to Quirin et al. (2009a), the IPANAT scales show relationships of small-to-moderate strength with explicit measures of the same affect type. Strengthening to some extent the concurrent validity of the IPANAT, we found a significant correlation between implicit positive affect and positive state affect as measured by the PANAS in our sample. The correlations we observed between IPANAT-PA and PANAS-PA (state and trait) are within the range of correlation coefficients reported by Quirin et al. (2009a, 2018). In our investigation, the IPANAT-NA did not correlate significantly with explicit measures of negative affect. The correlations found in our study between IPANAT-NA and PANAS-NA (state and trait) of 0.1 are low and can be interpreted as indicating a small effect size. However, they seem not to differ substantially from correlations observed in the previous research studies. In rather large samples from seven countries (Austria, China, Italy, Poland, Russia, USA, and Uzbekistan), correlations between IPANAT-NA and PANAS-NA (trait) varied between 0.16 and 0.28 (with a median *r* of 0.21; Quirin et al., 2018). Thus, the strength of the relationship between implicit and explicit negative affect appears to be in general rather weak (especially, when implicit affect is measured only once, and no previous affect induction is carried out).

Some limitations of our study have to be acknowledged. The number of included subjects is relatively low. Additionally, our sample consisting of young, well-educated women is not representative of the general population. This limits the generalizability of our results. Against this background, it seems necessary to replicate our findings in populations other than female college students. In future studies on the criterion validity of the IPANAT, it could be useful to administer real faces instead of schematic line drawings and examine whether implicit affect as assessed by the IPANAT also predicts perceived affect in these facial stimuli. The present investigation follows a traditional approach of test validation. According to this approach, validation is a process in which a variety of different forms of validity (such as criterion validity) are examined, and the test is set into a network of relations to outside criteria (Messick, 1989). Alternative modern forms of validation refer to Item Response Theory (IRT) models that link responses to items with underlying latent constructs through formalized statistical modeling (Geiser and Eid, 2010). IRT procedures, such as the Graded Response Model, have shown to improve positive and negative affect estimation (Zanon et al., 2016). IRT models have proven to be useful tools not only in the development of scales but also in the testing of theoretical assumptions (Lang and Tay, 2021). Future research studies on the convergent and discriminant validity of the IPANAT might benefit from administering latent multi trait-multimethod IRT models (see Runge and Lang, 2019, for an example of use in research on self-evaluation of implicit motives).

In sum, the present results support the validity of the IPANAT as a measure of implicit affect by showing differential relationships of the implicit positive and negative affect scales with perceptions of affect in facial expressions.

## Data Availability Statement

The raw data supporting the conclusions of this article will be made available by the authors, without undue reservation.

## Ethics Statement

The studies involving human participants were reviewed and approved by Ethikkommission, Medizinische Fakultät, Universität Leipzig. The participants provided their written informed consent to participate in this study.

## Author Contributions

VG, AK, and TS conceived and designed the experiment. VG collected the data. A-SW, MQ, TS, FS, and AK outlined the manuscript. A-SW, VG, and TS analyzed the data. A-SW wrote the manuscript with revisions and contributions from TS, VG, FS, AK, and MQ.

## Funding

This work was partially made possible through a grant from the Templeton Rlg. Trust (TRT 0119) supporting MQ.

## Conflict of Interest

The authors declare that the research was conducted in the absence of any commercial or financial relationships that could be construed as a potential conflict of interest.

## Publisher's Note

All claims expressed in this article are solely those of the authors and do not necessarily represent those of their affiliated organizations, or those of the publisher, the editors and the reviewers. Any product that may be evaluated in this article, or claim that may be made by its manufacturer, is not guaranteed or endorsed by the publisher.
